# Loss of *parla* Function Results in Inactivity, Olfactory Impairment, and Dopamine Neuron Loss in Zebrafish

**DOI:** 10.3390/biomedicines9020205

**Published:** 2021-02-18

**Authors:** Rawan Merhi, Michael Kalyn, Amanda Zhu-Pawlowsky, Marc Ekker

**Affiliations:** Department of Biology, Faculty of Science, University of Ottawa, Ottawa, ON K1N 6N5, Canada; rmerh088@uottawa.ca (R.M.); michael.t.kalyn@gmail.com (M.K.); azhup070@uottawa.ca (A.Z.-P.)

**Keywords:** Parkinson’s disease, *parla*, dopaminergic neurons, mitochondria, gene expression, locomotor, zebrafish

## Abstract

The presenilin-associated rhomboid-like (*PARL*) gene was found to contribute to mitochondrial morphology and function and was linked to familial Parkinson’s disease (PD). The *PARL* gene product is a mitochondrial intramembrane cleaving protease that acts on a number of mitochondrial proteins involved in mitochondrial morphology, apoptosis, and mitophagy. To date, functional and genetic studies of *PARL* have been mainly performed in mammals. However, little is known about *PARL* function and its role in dopaminergic (DA) neuron development in vertebrates. The zebrafish genome comprises two *PARL* paralogs: *parla* and *parlb*. Here, we established a loss-of-function mutation in *parla* via CRISPR/Cas9-mediated mutagenesis. We examined DA neuron numbers in the adult brain and expression of genes associated with DA neuron function in larvae and adults. We show that loss of *parla* function results in loss of DA neurons, mainly in the olfactory bulb. Changes in the levels of *tyrosine hydroxylase* transcripts supported this neuronal loss. Expression of *fis1*, a gene involved in mitochondrial fission, was increased in *parla* mutants. Finally, we showed that loss of *parla* function translates into impaired olfaction and altered locomotion parameters. These results suggest a role for *parla* in the development and/or maintenance of DA neuron function in zebrafish.

## 1. Introduction

Parkinson’s disease (PD) is the second most prevalent neurodegenerative disease, featuring motor signs that are often preceded by nonmotor symptoms such as anxiety/depression in 90% of PD patients and olfactory dysfunction [1]. Interestingly, recent data shows that more than 95% of patients with PD manifest significant olfactory loss [2]. Impairment in the sense of smell may precede motor symptoms by years, and thus can be used for the risk assessment of developing PD in asymptomatic individuals [2]. The main pathological feature of PD is the progressive and irreversible loss of dopaminergic (DA) neurons in the substantia nigra pars compacta of the midbrain [3]. Although the detailed etiology of PD remains unclear, most PD cases were found to be sporadic and can be associated with environmental factors [4]. Only 5–10% of patients result from familial PD [5]. With considerable effort in the past two decades, a number of genes associated with familial PD have been identified such as *PINK1, LRRK2, α-SYNUCLEIN, DJ-1, HTRA2*, and *ATP13A2* [6,7,8]. Interestingly, many of these genes are involved in regulating and maintaining mitochondrial function.

Mitochondria are dynamic organelles that undergo constant fusion and fission events to generate every cell’s energetic requirements [9,10]. Bioenergetically impaired mitochondria are rescued by fusion with the healthy mitochondrial network, whereas severely damaged mitochondria are segregated through fission and then degraded by mitophagy [11]. Several key proteins such as PINK1 and PARKIN have been found to play a major role in mitophagy [12] and dysregulation of the PINK1/PARKIN pathway has emerged as a disease mechanism for neurodegenerative disorders, most notably PD [13].

The presenilin-associated rhomboid-like protein (PARL), the orthologue of the mitochondrial rhomboid protease (rho7) found in *Drosophila,* regulates the PINK1/PARKIN mitophagy pathway in both mammals and *Drosophila* [8,14,15]. With mitochondrial dysfunction being common among PD patients, *PARL* is an excellent candidate as a gene associated with familial PD. In fact, researchers found a missense mutation in *PARL* in two patients manifesting PD [14]. To date, functional and genetic studies of *PARL* have been notably performed in mammals. However, little is known about *PARL* function and its role in DA development in vertebrates [16]. 

Zebrafish (*Danio rerio*) has emerged as a model for the study of movement disorders and neurodegenerative diseases. The neuronal circuitries involved in movement in zebrafish are well characterized, with essential molecular mechanisms being highly conserved and analogous to humans. Moreover, despite the remarkable anatomical differences between fish and mammals, it has been shown that more than 70% of all human disease genes have functional homologs in zebrafish [17]. Zebrafish models of PD have and continue to contribute to our better understanding of familial PD. 

In the adult zebrafish brain, DA neurons have been described in the telencephalon, diencephalon, olfactory bulb, pretectum, and preoptic area. It has been established that loss of DA neurons is associated with movement disorders [18], and it has been shown that DA neurons in the olfactory bulb (OB) carry sensory response to odor [19]. Despite notable differences, the zebrafish brain organization shows similarities to the human brain. In zebrafish, DA neurons are first detected at 18 h post-fertilization (hpf) in the ventral diencephalon (vDC). At 3 days post-fertilization (dpf), the central nervous system is well developed [20]. It has been previously suggested that clusters of DA neurons in the posterior tuberculum of the zebrafish brain vDC have ascending projections to the basal telencephalon of the subpallium and that these neurons are equivalent to the DA system found in the substantia nigra of the midbrain of mammals that have projections to the striatum [21]. However, Tay and colleagues [22] have recently identified an endogenous DA system in the subpallium that provides most of the local DA projections and that also connects to the vDC. It has been suggested that the observed DA neurons in the subpallium indicate that a predominant telencephalic dopamine source in zebrafish may be derived locally [22].

In zebrafish, two *parl* paralogs have been identified: *parla* and *parlb* [23]. Both *parl* transcripts were found to be expressed during embryonic development and in adult tissues. Morpholino-mediated knockdown of *parla* and/or *parlb* results in mild neurodegeneration supported by a decrease in DA cell numbers. In addition, the patterning of DA neurons is disrupted in the ventral diencephalon [23]. Interestingly, overexpression of zebrafish *pink1* leads to the rescue of larval mortality and DA neurons abnormalities, suggesting that similarly to mammals and *Drosophila*, *parl* is genetically upstream of *pink1* [23].

As *PARL* function remains to be fully characterized in zebrafish, particularly in the adult brain, we established a *parla* knockout (KO) line via CRISPR/Cas9 mediated mutagenesis. Here, we demonstrated how the loss of *parla* function results in a mild decrease in DA neuron cell number in the telencephalon and a severe neuronal loss in the olfactory bulb. This DA impact was supported through gene expression analysis in which *tyrosine hydroxylase 1* (*th1*) expression was severely reduced. These effects were shown to translate into behavior impairing phenotypes.

## 2. Material and Methods

### 2.1. Animal Care and Husbandry

All experiments were conducted using protocols approved by the University of Ottawa Animal Care Committee, and procedures were performed under the Animal Care and Veterinary Service guidance according to the Canadian Council for Animal Care ethical code BL-2081 (approved on 11 August 2020) Adult zebrafish were housed in circulating water systems at 28.5 °C under a 14 h light/10 h dark cycle. Embryos were obtained from the natural spawning of adult zebrafish. The embryonic stages were expressed in days post-fertilization (dpf) and adult stages in months post-fertilization (mpf).

### 2.2. CRISPR Design, sgRNAs Synthesis and Microinjections

A current zebrafish genome annotation (GRCZ11) in Ensembl was used for zebrafish gene coding and transcript information. For sgRNA design, CHOPCHOP software was employed [24]. Published protocol from Gagnon et al. (2014) [25] was followed to generate templates for sgRNA transcription by annealing gene specific oligonucleotides containing the T7 promoter sequence, the 20 base pairs (bp) target site (Table 1) followed by the PAM sequence, and a complementary region of 80 bp constant oligonucleotide. We synthesized three sgRNAs to be injected simultaneously to achieve multi-exon deletion. All sgRNAs were transcribed using the MAXIscript T7 kit (Life Technologies, Carlsbad, CA, USA), and the resulting RNA was purified using 5M ammonium acetate and ethanol precipitation. RNA bands were detected by electrophoresis on a 1% agarose gel and RNA purity and concentration were measured using NanoDrop 1000 spectrophotometer (ThermoFisher, Waltham, MA, USA). Zebrafish embryos were microinjected at the 1-cell stage. An injection solution was prepared as following: 40 ng/µL for each sgRNA, 20 ng/µL Cas9 protein (New England BioLabs, Ipswich, MA, USA), and 0.1% Phenol Red (Sigma, Burlington, MA, USA). Dead and deformed fish embryos were removed, and healthy ones were raised to adult fish as F_0_ founders.

### 2.3. Description of parla Mutation

**Figure 1:**
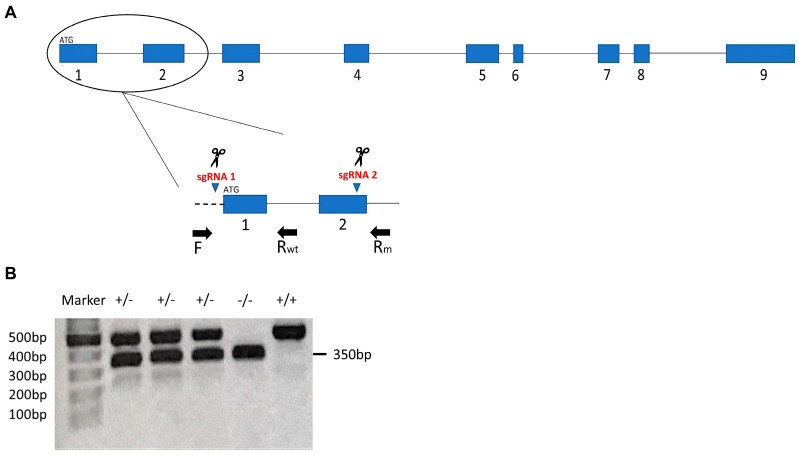
Exon 1 and 2 deletion in the *parla* gene by CRISPR/Cas9-mediated mutagenesis. (**A**) Schematic representation of sgRNA targeting and deletion of *parla* exons 1 and 2. The two sgRNAs that contributed to the deletion are shown. Exons are represented with blue cylinders and introns with black lines between the exons. The dashed line represents the 5′ UTR region. Primers used in genotyping are represented by F (forward primer), Rwt (reverse primer for the WT), and Rm (reverse primer for the mutant). (**B**) Agarose gel showing WT band (500 bp) and mutant band (350 bp). The marker used is a 10 kb DNA ladder mix (GeneRuler^TM^). +/+ refers to WT. +/− and −/− refer to heterozygous and homozygous mutants, respectively.

Two of the three simultaneously injected sgRNAs resulted in the deletion of 1.4 kilobases (kb) corresponding to the region that spans exon 1 to exon 2 (EX1_2del according to the Nomenclature for the description of sequence variations [26]) in the genomic DNA sequence of *parla* gene (Figure 1A). Based on the Zebrafish Information Network (ZFIN) nomenclature conventions, we named the resulting mutant line *parla ^ot510^*. Genomic DNA was extracted from fins followed by PCR amplification using forward (F) and reverse (Rm) primers that flank the target sites to amplify the mutant band and another reverse primer (Rwt) that binds to the region between the target sites to amplify the WT band (primer sequences are listed in Table 2). PCR products for the mutant and WT were separated by gel electrophoresis on a 1% agarose gel and sizes of the resulting bands were 500 bp for wild type and 350 bp for the mutant (Figure 1B). Deletion was further confirmed by Sanger sequencing and sequencing data showed that deletion junctions resulted from the precise ligation of the blunt-ended double-strand breaks (DSBs) created by Cas9; each DSB occurred exactly 3 bp upstream of the PAM sequence (data not shown). Based on cDNA sequencing data for the *parla* gene previously performed by Noble and colleagues [23] and based on Ensembl database, we obtained the cDNA sequence following deletion, then we determined the potential protein sequence using Expasy database (Swiss Bioinformatics Resource Portal). A deletion from the amino acid Methionine 1 to Arginine 71 (M1_R71del [26]) is predicted to have occurred.

### 2.4. Collection of Zebrafish Brain Tissue 

Zebrafish brain tissue was collected from both male and female fish and results were pooled together as no gross morphological differences were observed between brains of the two sexes. Adult fish were euthanized on ice at an age from 7 to 12 months post fertilization (mpf) in system water followed by immediate decapitation. The whole head was fixed in 4% PFA/PBS overnight at 4 °C. After dissection, brains were placed for additional 20 min in PFA for post-fixation. The tissue was washed with PBS and then immersed in 30% sucrose/PBS overnight at 4 °C. Whole brains were incubated in a solution of 1:2 30% Sucrose: OCT Compound (Tissue-Tek, VWR Canada) for 10 min, placed in cryomolds, and frozen in liquid nitrogen. Cryosections of 18 μm were obtained with a CM3050S cryostat (Leica, Concord, ON, Canada) in quadruplicate slides.

### 2.5. Histology and Immunohistochemistry (IHC)

Sections were first allowed to rest for 30 min at room temperature, and then an antigen retrieval protocol was performed. Sections were treated for 20 min at 85 °C in 0.01M sodium citrate/0.05% Tween-20 solution and cooled down to room temperature (RT) for 15 min prior to blocking. Sections were then rehydrated in PBST (PBS with 0.1% Tween-20) and blocked in 10% fetal bovine serum in PBST for 2 h at RT. Primary antibody (mouse anti-TH, Temecula, CA, USA; Cat No. AB318) diluted 1:450 was used according to the manufacturers’ instructions and protocol optimization. The primary antibody incubation was carried out overnight at 4 °C in 1% fetal bovine serum in PBST. Sections were then washed 3× for 5 min with PBST and incubated in the dark for 2 h at room temperature with the secondary antibody (goat anti-mouse IgG Alexa 488; Thermo Fisher Scientific, Cat No. A-11001) diluted 1:1000. Slides were finally washed in PBST and mounted using Vectashield mounting media (Vector Labs, Burlington, ON, Canada). Images were acquired with either an Olympus FV1000 Confocal microscope or a Zeiss AxioPhot Fluorescence Microscope. Acquired images were processed using Olympus Fluoview Software or ImageJ. Cell counts were obtained from Z-stacking images acquired from several planes to form a composite 3-Dimentional (3D) set of data for each tissue sample. The images combined at different focus distances give a resulting image with a greater depth of field than any of the individual source images. Four to 5 tissue samples of comparable planes and areas were counted throughout the olfactory bulb (OB) and telencephalon and were averaged for each fish. Relative TH+ cell counts were calculated by comparing the summed average number of TH+ cells with WT. Confocal images show representative single planes. 

### 2.6. RNA Isolation, cDNA Synthesis and qRT-PCR

Total RNA was extracted from the dissected whole brain of each adult fish using homogenization with TriZol (InVitrogen, ThermoFisher, Waltham, MA, USA) according to manufacturer’s protocol. A total of three brains were isolated from adult fish. The same homogenization technique and protocol were used for total RNA extraction from pooled 5 dpf larvae. Total RNA was extracted from three biological replicates, each containing 7-pestle homogenized whole larvae. Concentration and purity of extracted RNA were obtained using NanoDrop 1000 spectrophotometer (ThermoFisher, Waltham, MA, USA). Synthesis of cDNA was accomplished using the iScript^TM^ cDNA Synthesis Kit (Life Science Research, Bio-Rad, St Laurent, QC, Canada) following the manufacturer’s protocol. qRT-PCR reactions were constituted of 5 μL SsoFast^TM^ EvaGreen^®^ Supermix (Bio-Rad), 0.4 μL forward primer, 0.4 μL reverse primer, 0.2 μL nuclease-free water, and 4 μL cDNA. Reactions were performed in triplicates using the Bio-Rad CFX96 instrument. Normalized quantification of the number of *parla*, *parlb*, *th1*, *dat*, *opa1*, *pink1*, *mfn1*, and *fis1* transcripts was achieved through the comparative Cq method using three reference genes: *Beta-Actin (β-Act)*, *ribosomal protein l13a (rpl13a)*, and *elongation factor 1 alpha (ef1a)*. Oligonucleotide primers’ sequences are listed in Table 3.

### 2.7. Swimming Activity

The effects of DA neuron loss on motor function were addressed in 7 to 12 mpf adult zebrafish. Fish were allowed to acclimate by swimming freely for a 2-min period in individual static tanks, followed by a 5-min recording period. The parameters analyzed for behavioral assessment were total distance travelled by the fish, average velocity, and inactivity duration. A sample size of 14 adult zebrafish was chosen and swimming activity was recorded using the ZebraLab software and the ZebraCube tracking system (ViewPoint Life Science, Lyon, France). The tracking system consists of LED lights, infrared illumination, and a mounted camera for swimming recording under dark and light conditions.

### 2.8. Olfactory Function

Olfactory function was tested according to Godoy et al. 2020 [19]. Briefly, tanks used for this experiment were customized and contained a mid-tank division which divided the tank into a neutral zone, and left and right arms. Seven to 12 mpf adult zebrafish were used with a sample size of 7 fish. Each animal was allowed to swim freely in the tank for 2 min prior to the addition of the repulsive stimulus cadaverine (Sigma-Aldrich) to the arm where the fish was located. The time spent in each area was recorded for another 3 min. This elapsed time was sufficient to allow the stimulus to diffuse throughout one of the tank’s arms without reaching a different tank area. The ratio of time spent in the stimulus arm was calculated post-stimulus by dividing the percent of time spent in stimulus by the total recording time. Cadaverine (80 μL of 1 mM stock solution) was delivered with a micropipette into the rearmost portion of the arm. Control animals received the same volume of system water.

### 2.9. Statistical Analysis

Statistical analysis was performed using the software GraphPad Prism v.7 (San Diego, CA, USA). DA neuron loss was analyzed using a two-way ANOVA followed by Tukey’s multiple comparison test. Gene expression data were established using a multiple *t*-test comparison followed by Holm-Sidak method to determine significance. The total distance, average velocity, and inactivity duration were analyzed using a one-way ANOVA followed by Dunnett’s multiple comparison test. Zebrafish olfactory function statistical power was assessed using multiple comparison *t*-test with Holm–Sidak post-hock correction. Data are shown as the mean ± standard error of the mean (SEM) or as the median with 95% confidence intervals (CI). When the number of data points (*n*) is lower than 10, only the data and median are shown. When *n* > 10, error bars are included [28]. Statistical significance was determined when *p*-value < 0.05 and was indicated as * *p* < 0.05, ** *p* < 0.01, *** *p* < 0.001.

## 3. Results

Using CRISPR/Cas9-mediated mutagenesis, we generated a multi-exon deletion in the *parla* gene. Based on the Ensembl Genome Database, the *parla* gene comprises a total of 9 exons (Figure 1A). By injecting 3 sgRNAs simultaneously in zebrafish embryos at the 1-cell stage, we were able to create deletion of the first two exons in the *parla* gene. Fish that are homozygous for the *parla* mutation were obtained through genotyping and found to be both viable and fertile. Moreover, we did not notice any obvious gross morphological or behavioral defects in the mutants. 

The various measurements shown in our study were performed in homozygous (*parla ^ot510^*) and heterozygous (*parla ^+/ot510^*) fish and compared to WT (*parla ^+^*) controls.

### 3.1. Gene Expression Analysis

#### 3.1.1. Adult Brain

To assess the effect of *parla* KO on gene expression in the adult zebrafish brain, we determined expression of genes such as *PTEN-induced kinase 1* (gene: *pink1*; protein: PINK1) and *optic dominant atrophy* (gene: *opa1*; protein: OPA1), both of which encode for mitochondrial proteins known to be PARL substrates [8,29]. Additionally, we investigated whether *tyrosine hydroxylase 1* (gene: *th1*; protein: TH) and *dopamine transporter* (gene: *dat*; protein: DAT) correlated with the observed decreases in DA neuron cell numbers. Lastly, we checked whether expression of genes involved in mitochondrial function and dynamics is affected by analyzing expression of *mitochondrial fission 1* (gene: *fis1*; protein: FIS1) and *mitofusin 1* (gene: *mfn1*; protein: MFN1). 

**Figure 2:**
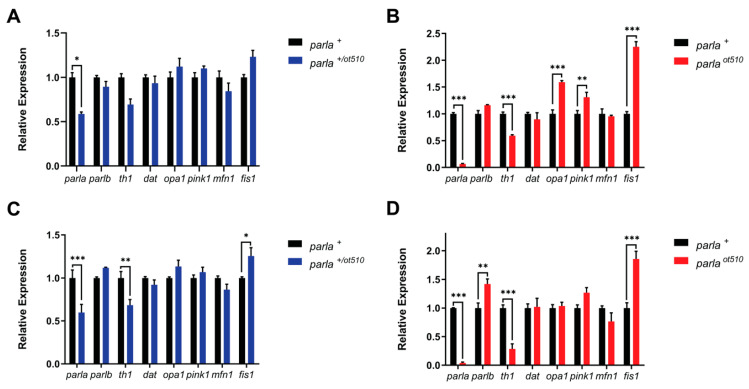
Effect of *parla* loss-of-function on the expression of genes associated with dopaminergic- and mitochondrial function in larvae and in the adult zebrafish brain. (**A**,**B**) Relative expression of *parla*, *parlb*, *th1*, *dat*, *opa1*, *pink1*, *mfn1*, and *fis1* of 5 dpf zebrafish larvae (*n* = 3. Each replicate contains 7 homogenized whole larvae). (**C**,**D**) Relative expression of *parla*, *parlb*, *th1*, *dat*, *opa1*, *pink1*, *mfn1*, and *fis1* of 4–10 mpf adult zebrafish brains (*n* = 3). WT fish are represented by *parla ^+^*, homozygous mutants by *parla ^ot510^*, and heterozygous mutants by *parla ^+/ot510^*. Bars represent the Mean ± the SEM. * (*p* < 0.05), ** (*p* < 0.01), *** (*p* < 0.001).

Loss of the *parla* gene was confirmed by a 96% decrease in *parla* expression levels seen in the homozygous mutants (*parla ^ot510^*) (Figure 2D). In addition, fish heterozygous for the mutation (*parla ^+/ot510^*) showed a 60% reduction in *parla* transcript levels (Figure 2C). As for *parlb* expression, there was a 42% increase in *parla ^ot510^* mutants which might indicate the existence of a compensatory mechanism for the two paralogous genes *parla* and *parlb* (Figure 2D). Similarly, there was a 12% increase in *parlb* expression observed in *parla ^+/ot510^* mutants (Figure 2C). Interestingly, *parla ^+/ot510^* and *parla ^ot510^* animals exhibited 30% and 70% decreases in the expression of *th1*, respectively (Figure 2C,D). However, there was no change in the expression of the *dat* gene. Similarly, *opa1* showed no change in expression. A slight 27% and 7% upregulation in *pink1* expression was observed in *parla ^ot510^* and *parla ^+/ot510^* individuals, respectively. A slight 24% and 14% decrease in the expression of *mfn1* were seen in both *parla ^ot510^* and *parla ^+/ot510^* zebrafish, respectively. Finally, the expression of *fis1* was observed to be increased by 25% and 86% in *parla ^+/ot510^* and *parla ^ot510^* animals, respectively (Figure 2C,D). As *fis1* encodes for a protein that plays a role in mitochondrial fission, this change in expression might suggest increased mitochondrial fission events in *parla* KO fish.

#### 3.1.2. Larvae

Knowing that *parla* is expressed ubiquitously [23], we analyzed gene expression throughout the entire body of larvae. The most noticeable changes in expression were seen in *parla ^ot510^* larvae for *th1*, *opa1*, *pink1*, and *fis1* genes. There were 31% and 41% decreases in *th1* expression in both *parla ^+/ot510^* and *parla ^ot510^* larvae, respectively (Figure 2A,B). However, statistical significance was not reached for *parla ^+/ot510^* larvae. This decrease in expression is in line with the decrease in *th1* expression observed in the brain of *parla ^ot510^* adults. In addition, there were significant increases of 60% and 31% in *opa1* and *pink1* expression, respectively, in *parla ^ot510^* larvae (Figure 2B). Similarly, *opa1* and *pink1* expression was increased by 12% and 10%, respectively, in *parla ^+/ot510^* larvae, but this did not reach statistical significance (Figure 2A). The highest increases in expression were observed for *fis1* in *parla ^ot510^* larvae, which reached 2.25 times the levels measured in WT (*parla ^+^*) controls (Figure 2B). In *parla ^+/ot510^* larvae, *fis1* expression showed a 23% increase, but this did not reach statistical significance (Figure 2A). These increases in *fis1* expression are in line with those seen in the brain of adult *parla ^ot510^* fish. Finally, the expression levels of *parlb*, *dat*, and *mfn1* genes did not change significantly compared to WT controls (Figure 2A,B).

### 3.2. DA Neuronal Loss in the Telencephalon and Olfactory Bulb

**Figure 3:**
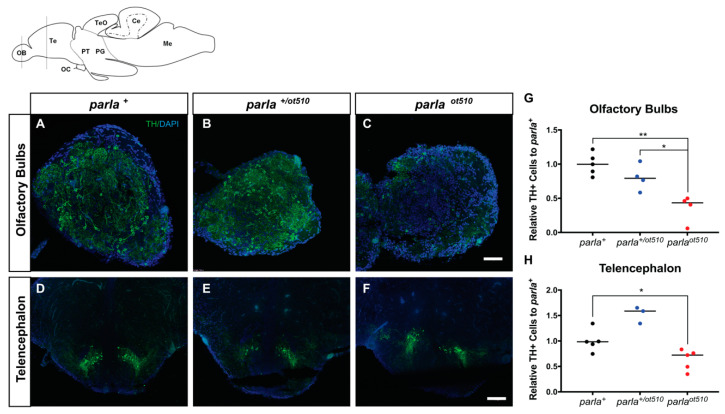
Impacts of *parla* loss-of-function on dopaminergic (DA) neuron cell number in the telencephalon and olfactory bulbs of adult zebrafish. Schematic representation of olfactory bulb (OB) and telencephalon sections depicted in the top left panel. (**A**–**F**) Transverse cryosections showing immunofluorescence of TH+ (Green) and DAPI stained (Blue) cells in dissected OB (**A**–**C**) and telencephalon (**D**–**F**) of adult zebrafish. Scale bar: 50 µm. (**G**,**H**) Quantification of the number of cells expressing TH. WT fish are represented by *parl* ^+^, homozygous mutants by *parla ^ot510^*, and heterozygous mutants by *parl ^+/ot510^* (*n* = 5 for *parla ^+^*, *n* = 4–5 for *parla ^ot510^* and *n*= 3–4 for *parla ^+/ot510^* adult zebrafish). Bars represent the Median. * (*p* < 0.05), ** (*p* < 0.01).

To determine the effect of *parla* loss-of-function on DA neuron number and patterning, we performed IHC assays in adults ranging from 7 to 12 mpf in both the telencephalon and olfactory bulb regions using TH1 antibody which is widely used for the detection of DA neurons [30]. A reduction in TH positive (TH+) cell number reflects loss of DA neurons. Numbers of TH+ cells for both *parla ^ot510^* and *parla ^+/ot510^* mutants were compared to WT (Figure 3A,D). TH+ cell reductions of 60% and 20% were observed in the olfactory bulbs (OB) for both *parla ^ot510^* and *parla ^+/ot510^* animals, respectively (Figure 3B,C,G). Similarly, a mild reduction of 25% in TH+ cells was seen in the telencephalon for *parla ^ot510^* mutants (Figure 3F,H). These reductions corroborate the previously observed decrease in *th1* expression level. On the other hand, there was no observable decrease in TH+ cell number in the telencephalon for *parla ^+/ot510^* mutants (Figure 3E).

Interestingly, we observed mis-patterning of TH+ cells notably at the level of axonic projections in the telencephalon of *parla ^ot510^* fish (R. Merhi, M. Kalyn, A. Zhu-Pawlowsky and M. Ekker, University of Ottawa, Ottawa, ON, Canada, 2021, unpublished observations).). This observation is consistent with previous studies in which patterning of DA neurons was found to be perturbed in the vDC of *parl* morphants [18,23].

### 3.3. Effects on Olfactory Function

**Figure 4:**
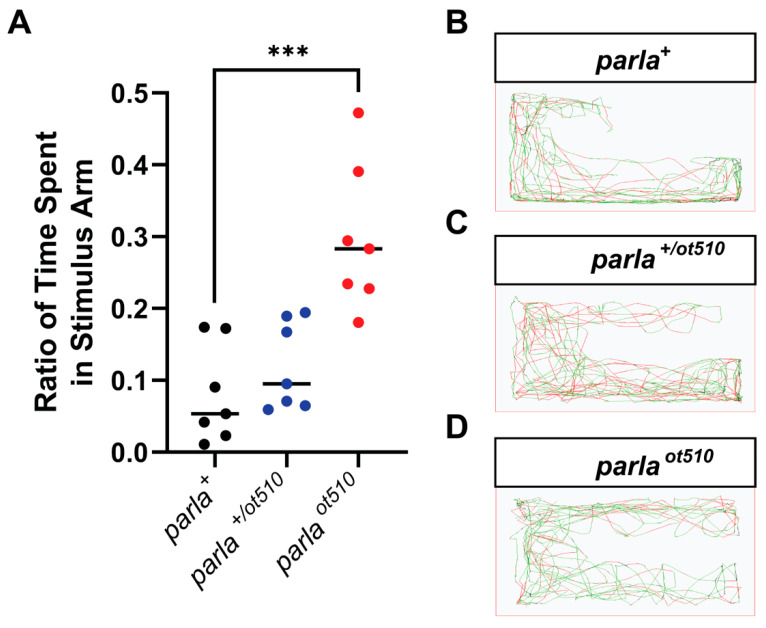
Decreased olfactory function in adult zebrafish with *parla* loss-of-function. (**A**) Ratio of time spent in stimulus arm of 7–12 mpf adult zebrafish (*n* = 7). (**B**–**D**) Path images of a representative for WT fish (*parla ^+^*), homozygous mutants (*parla ^ot510^*), and heterozygous mutants (*parla ^+/ot510^*). Bars represent the Median. *** (*p* < 0.001).

To determine if the loss of DA neurons in the OB of adult mutants has implications on olfactory function, we performed a repulsive stimulus test. The repulsive stimulus used in this experiment is cadaverine, which was administered to the arm of an apparatus in which the fish was acclimatized [19]. The time spent in each section of the tank was then recorded and the ratio of time spent in stimulus arm was calculated. Interestingly, *parla* *^ot510^* fish spent on average of more than 3.5 times of the recorded period in the stimulus arm where cadaverine was added compared to the WT zebrafish (Figure 4A,B,D). On the other hand, there was a 48% increase in the ratio of time spent in the stimulus arm observed in *parla* *^+/ot510^* mutants (Figure 4A,C). 

### 3.4. Effects on Locomotion

**Figure 5:**
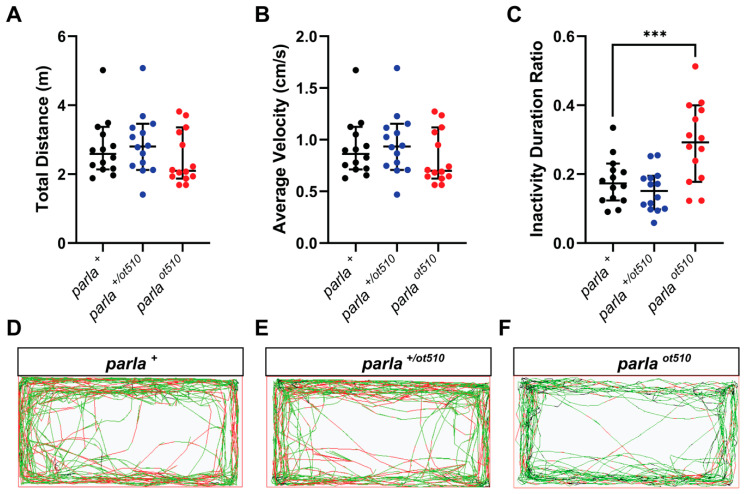
Swimming activity of adult mutant and WT zebrafish. (**A**–**C**) Impact of *parla* loss-of-function on total swim distance, average swimming velocity and inactivity periods (*n* = 14 adult zebrafish). (**D**–**F**) Path images of a representative for WT fish (*parla ^+^*), homozygous mutants (*parla ^ot510^*), and heterozygous mutants (*parla ^+/ot510^*). Bars represent the Median with 95% CI. *** (*p* < 0.001).

To further investigate whether the decrease seen in DA cell number in the brain of *parla* mutants translates into any behavior impairing phenotype, we analyzed locomotor activity in adult zebrafish. Interestingly, the total distance travelled by the fish and average velocity were not significantly affected in both *parla* *^ot510^* and *parla* *^+/ot510^* fish (Figure 5A,B). However, there was an 11% decrease in distance travelled and average velocity in *parla* *^ot510^* mutants. Interestingly, *parla* *^ot510^* fish showed a 62% increase in inactivity duration as compared to WT (Figure 5C). In contrast, no significant impact on inactivity was observed in heterozygous (*parla* *^+/ot510^*) animals. Furthermore, distance travelled, average velocity, and inactivity duration were not affected in 5 dpf larvae (R. Merhi, M. Kalyn, A. Zhu-Pawlowsky and M. Ekker, University of Ottawa, ON, Canada, 2021, unpublished observations).

## 4. Discussion

To date, the vast majority of studies addressing *PARL* function have been performed in yeast, *Drosophila*, and mammals [29,31,32]. It is only recently where the *PARL* paralogues have been identified in a zebrafish model [23]. This evolutionary conservation of *PARL* is evidence of the critical role that this protease plays across the animal kingdom. 

In yeast and *Drosophila*, *rhomboid 1* and *rhomboid-7* (the *PARL* orthologues, respectively), possess critical roles in mitochondrial maintenance via fusion and fission regulation [29,31]. However, the loss-of-function of *rhomboid-7* in *Drosophila* rendered complete lethality within a population by 3 dpf [29]. In mammals, targeted *PARL* null mice show progressive muscle and immune organ atrophy with death occurring before the age of three months [16]. Moreover, severe apoptosis accompanied with mitochondrial morphological changes at the level of the cristae occurred in the fibroblasts and liver of *PARL* KO mice suggesting that *PARL* plays an anti-apoptotic role by regulating cristae morphology [16]. Similarly, in a recent study, morphological abnormalities in mitochondria were detected in the brain, indicating that the accumulation of these mitochondrial structural and functional defects was correlated to the significant neurodegeneration observed in *Parl ^−/−^* mice [32].

In zebrafish, the transcripts of the two *parl* paralogues identified were shown to be expressed during embryonic development and in adult tissues. Morpholino-mediated knockdown of both *parla* and *parlb* genes resulted in embryonic lethality, increased cell death, and mild neurodegeneration of DA neurons [23]. Interestingly, the Parla protein showed a 67% amino acid identity to human PARL, higher than for Parlb (55%). Moreover, it was suggested that in zebrafish, proper DA neuron development depends particularly on *parla* rather than *parlb* function [23]. These results were observed in 1 to 3 dpf zebrafish larvae following a morpholino-mediated knockdown of *parl* genes. However, given the transient nature of gene-knockdown phenotypes, additional loss-of-function studies in larvae and adult zebrafish needed to be performed.

In our study, we generated a permanent multi-exon deletion in the *parla* gene via CRISPR/Cas9-mediated mutagenesis, and we found that, similarly to what was shown in the morpholino study in larvae [23], loss-of-function of the *parla* gene leads to severe DA neuron loss and mis-patterning in the adult zebrafish brain. This was shown by the reduced number of TH+ cells in both the telencephalon and olfactory bulbs. We also observed disorganized axonal tracts of DA neurons in the olfactory bulbs (R. Merhi, M. Kalyn, A. Zhu-Pawlowsky and M. Ekker, University of Ottawa, ON, Canada, 2021, unpublished observations). Tyrosine hydroxylase (TH) is the rate-limiting enzyme in dopamine biosynthesis and an important marker of catecholaminergic neurons. It is commonly used in studies to label DA neurons [30]. Two genes, *th1* and *th2*, encode the TH enzyme in zebrafish, and both Th1 and Th2 proteins are highly similar to the mammalian TH [33]. Immunohistochemical analysis of Th1 was performed as it is widely expressed in all brain regions, whereas Th2 is localized to hypothalamic, posterior tubercular, and preoptic regions of the zebrafish brain [34]. In two of the sensory and behavioral epicenters of the brain, the telencephalon and olfactory bulbs, we observed considerable reductions in Th1 at both the mRNA and protein level in *parla ^ot510^* adult zebrafish. Intriguingly, there was no change in the *dopamine transporter (dat)* transcript levels in both mutant adult brains and in mutant larvae. This could be explained by a possible upregulation of *dat* in the DA neurons that survive. Loss of *parla* function might be affecting dopamine biosynthesis, although DA neurons that survive are still expressing *dat* for dopamine re-uptake. 

Interestingly, the severe DA neuron loss seen in the olfactory bulbs translates into an olfactory impairment observed in the repulsive stimulus test. However, the locomotor assessment showed that there were no remarkable effects on the adult fish’s ability to perform locomotor functions such as total distance travelled and velocity. This could possibly be explained by the considerable number of TH+ cells that remain residing within the telencephalon of the *parla* mutant fish. The telencephalic region has been previously characterized with DA clusters and projections claimed to be analogous to the nigrostriatal pathway in humans involved in movement modulation [35]. Therefore, it is unlikely that the inability of fish to swim away of the repulsive stimulus in the olfactory test could be due to locomotor impairments, but are rather due to the inability to detect or respond to the stimulus. It is possible that impaired olfaction precedes impaired locomotion in zebrafish as seen in human PD patients that manifest locomotor dysfunction after losing the ability to smell [2,36]. Intriguingly, mutant fish showed high levels of inactivity periods. We hypothesize that the inactivity episodes noted are not due to muscle dysfunction, as distance travelled and average velocity of the fish were not affected, but are rather due to the mild DA neuron reduction observed in the telencephalic region. 

Striking mitochondrial functional and morphological abnormalities such as fragmentation and aggregation have been observed in yeast, *Drosophila*, and mice with a loss-of-function mutation in the *PARL* gene [16,29,31,32]. Moreover, it has been suggested that mitochondrial damage, likely due to an increase in reactive oxygen species, results in depolarization of the inner mitochondrial membrane and loss of membrane potential which induces mitochondrial fragmentation [36]. In our study, we analyzed expression of the *fis1* gene involved in mitochondrial fission. We observed a significant increase in *fis1* mRNA expression in both mutant adult brains and in mutant larvae. We hypothesize that mitochondria of adult fish brains and larvae with a loss-of-function in *parla* gene undergo increased mitochondrial fission turnover due to mitochondrial dysfunction. A more concrete observation such as live mitochondrial imaging is still needed to confirm this hypothesis.

Robust evidence from previous studies indicates that PINK1, a mitochondrial kinase with a key role in regulating mitophagy, is a substrate of PARL in mammals and other organisms [8,23,37,38]. This kinase was also found to play a critical role in the regulation of complex I activity in mitochondria [39] and to be implicated in the pathogenesis of PD. The absence of PINK1 cleavage by PARL results in the accumulation of PINK1 on the outer mitochondrial membrane followed by induction of the ubiquitination event that promotes the clearance of defective mitochondria [37]. Similarly to other organisms, the *pink1* gene was found to genetically function downstream of *parl* as part of a conserved pathway in vertebrates [23]. Intriguingly, our gene expression analysis shows that *pink1* expression was not significantly affected in adult brain tissue following loss of *parla* gene function. In fact, Noble and colleagues have shown that lack of both Parl proteins in larvae can be rescued by the expression of either zebrafish or human PINK1 and have suggested that compensatory mechanisms might be involved in Pink1 cleavage in zebrafish, despite the fact that Pink1 is a substrate of Parl [23]. Moreover, using RNA interference, previous findings showed that three other mitochondrial proteases affected levels of PINK1 in HEK293T cells [40]. In contrast, we found that *pink1* expression was significantly increased in *parla ^ot510^* larvae. Similar observations were noted for *opa1*, whose expression was significantly increased in *parla ^ot510^* larvae, but not in the mutant adult brains. In *Drosophila*, yeast, and mice, a genetic interaction between PARL and OPA1 seems to be established, although whether OPA1 is a substrate of PARL is still debatable. Given the differential *pink1* and *opa1* gene expression profiles seen in zebrafish mutant larvae versus adult mutant brains, we speculate that loss-of-function of *parla* in zebrafish might have different impacts on *pink1* and *opa1* gene expression in a tissue or developmental stage-dependent manner. The lack of significant effects on the expression of *pink1*, *opa1*, or of other genes in *parla ^ot510^* adult brains or larvae might also be explained by possible compensatory mechanisms exerted by the paralogous *parlb* gene in zebrafish. In fact, *parlb* expression was found to increase following the loss-of-function of *parla* in both adult brains and larvae. Morpholino-knockdown of *parla* and *parlb* simultaneously was shown to induce larval mortality. In contrast, loss-of-function of one of the two genes did not result in increased mortality [23]. Similarly, we found that *parla ^ot510^* fish did not present significant larval mortalities and were viable well into adulthood. In mice, loss of *Parl* function was found to be lethal [16]. Thus, functional redundancy of the zebrafish *parl* paralogs confers some advantages to this model organism [41].

## 5. Conclusions

Overall, we have shown through targeted CRISPR/Cas9-mediated loss-of-function of the *parla* gene, its importance for DA neuron number and patterning in the telencephalon and olfactory bulb of the adult zebrafish brain. We also suggested that loss of DA neurons is correlated with the behavioral and olfactory impairments observed in the adult fish. Given the prevalence of mitochondrial dysfunction in PD pathologies and the role of *PARL* in mitochondrial homeostasis, the generation of *PARL* zebrafish mutant lines is beneficial in the understanding of the specific function played by *PARL* on the onset of PD and could lead to the development of novel therapeutic approaches and strategies.

## Figures and Tables

**Table 1:** List of target sequences of *parla* gene for sgRNAs generation.

Region Targeted	Target Sequence	Orientation
5′ UTR	GGCGCGCTGTGAGCCGGAAG	5′-3′
5′ UTR	GGAAAGCGCAGTTTTATGCA	5′-3′
Exon 2	GCCAAATGACTTGGTGGGCC	3′-5′

**Table 2:** List of primers designed for PCR amplification.

Primer	Sequence (5′-3′)
F	CTCCAACAGGAGCCGCTATG
Rm	CTAGGGATTTTGTGCATGCTTAC
Rwt	ATTCATTATCCTGACAGGCC

**Table 3:** List of primers designed for qRT-PCR.

Primer	Forward Sequence (5′-3′)	Reverse Sequence (5′-3′)
*parla*	GTGTTTTGTTGTTGGCGGGT	ATGTAGACAGCAGCATCGG
*parlb*	ATCACGCAGCACATCTTCCT	TATTAGTGGCTCGCGCTTCC
*th1*	GACGGAAGATGATCGGAGACA	AGAAATCGGAACATGGCGG
*dat*	AGACATCTGGGAAGGTGGTG	ACCTGAGCATCATAGAGGCG
*opa1*	GCTTGAGCCCTTGGAAAAGGAA	TGGCAGGTGATCTTGAGTGTTGT
*pink1*	GGCAATGAAGATGATGTGGAAC	GGTCGGCAGGACATCAGGA
*mfn1*	CTGGGTCCCGTCAACGCCAA	ACTGAACCACCGCTGGGGCT
*fis1*	CCCTGAACCTTCCAGTGTTT	GTCTCTGGAAACGGGTCCTT
*β-Act* ^1^	CGAGCTGTCTTCCCATCCA	TCACCAACGTAGCTGTCTTTCTG
*rpl13a* ^1^	TCTGGAGGACTGTAAGAGGTATGC	AGACGCACAATCTTGAGAGCAG
*ef1a* ^1^	CTGGAGGCCAGCTCAAACAT	ATCAAGAAGAGTAGTACCGCTAGCATTAC

^1^ Reference: Tang et al. (2007) [27].

## Data Availability

Data is contained within the article.
